# Diverse Bone Morphogenetic Protein Expression Profiles and Smad Pathway Activation in Different Phenotypes of Experimental Canine Mammary Tumors

**DOI:** 10.1371/journal.pone.0007133

**Published:** 2009-09-22

**Authors:** Helena Wensman, Nils-Erik Heldin, Gunnar Pejler, Eva Hellmén

**Affiliations:** 1 Department of Anatomy, Physiology and Biochemistry, Swedish University of Agricultural Sciences, Uppsala, Sweden; 2 Department of Genetics and Pathology, Uppsala University, Uppsala, Sweden; Katholieke Universiteit Leuven, Belgium

## Abstract

**Background:**

BMPs are currently receiving attention for their role in tumorigenesis and tumor progression. Currently, most BMP expression studies are performed on carcinomas, and not much is known about the situation in sarcomas.

**Methodology/Principal Findings:**

We have investigated the BMP expression profiles and Smad activation in clones from different spontaneous canine mammary tumors. Spindle cell tumor and osteosarcoma clones expressed high levels of BMPs, in particular BMP-2, -4 and -6. Clones from a scirrhous carcinoma expressed much lower BMP levels. The various clones formed different tumor types in nude mice but only clones that expressed high levels of BMP-6 gave bone formation. Phosphorylated Smad-1/5, located in the nucleus, was detected in tumors derived from clones expressing high levels of BMPs, indicating an active BMP signaling pathway and BMP-2 stimulation of mammary tumor cell clones *in vitro* resulted in activation of the Smad-1/5 pathway. In contrast BMP-2 stimulation did not induce phosphorylation of the non-Smad pathway p38 MAPK. Interestingly, an increased level of the BMP-antagonist chordin-like 1 was detected after BMP stimulation of non-bone forming clones.

**Conclusions/Significance:**

We conclude that the specific BMP expression repertoire differs substantially between different types of mammary tumors and that BMP-6 expression most probably has a biological role in bone formation of canine mammary tumors.

## Introduction

Bone morphogenetic proteins (BMPs), members of the TGF-β superfamily, constitute a group of extracellular factors that are important in many cellular processes. Originally they were named due to their ability to induce bone formation [1], but it is now well recognized that BMPs can participate in numerous other processes [2]. To date, approximately 15 BMPs have been identified and characterized [3]. The BMPs can be divided into two subclasses, with BMP-2 and -4 belonging to one subclass and BMP-5, -6, -7, and -8 to another [4]. BMPs signal via type I and -II cell surface receptors [5] and the signal is transduced via phosphorylation of Smad-1, -5 and -8 proteins, followed by nuclear translocation of the phosphorylated Smad [6].

The different BMPs have distinct functions during development [7]. For example, when the osteogenic activity of 14 types of BMPs was studied *in vivo*, BMP-6 and -9 induced a more robust and mature ossification than the others [8]. Although BMPs have important functions during physiological bone formation [3], [9], BMPs are also implicated in the formation of bone tumors [10]. Further, there is currently an interest for BMPs in relation to mammary tumors, [11], [12] and metastases derived from human breast cancer often localize to bone [13], whereas this is uncommon in dogs [14], [15]. Moreover, it is known that mixed mammary tumors of canine origin often are associated with bone formation[14].

In a recent study, the expression of BMPs and BMP receptors in human mammary carcinoma was studied [12]. However, the expression of BMPs in mammary tumors of non-epithelial origin, e.g. osteosarcoma or spindle cell tumors, have not been studied. Further, previous investigations relating to the role of BMPs in tumorigenesis have mostly focused on a limited number out of the various BMPs. Moreover, the functionality of the BMP pathway in tumor tissue has not been studied extensively. Finally, the possibility that the BMP expression pattern is tumor phenotype-specific has not been addressed. In this study we addressed all of these issues.

## Materials and Methods

### Cell lines

The cell line CMT-U353B, established from a poorly differentiated combined canine mammary osteosarcoma was used together with cell line CMT-U353H4 established from a canine mammary simple scirrhous carcinoma [16]. We also used the cell line CMT-U309, established from a canine mammary spindle-cell tumor [17]. The cell lines were cloned (after passage 16, CMT-U353 H4; passage 22, CMT-U353 B; passage 15, CMT-U309) by seeding approximately 30 cells per 10 cm cell culture dish. Cell colonies were transferred to new plates and further cultivated. The cell lines and clones were cultured in RPMI 1640 medium (Invitrogen, Carlsbad, CA), supplemented with 2 mM L-Glutamine (SVA, Uppsala, Sweden), 10% fetal bovine serum (Invitrogen, Carlsbad, CA), penicillin (120 µg/ml) and streptomycin (100 µg/ml; SVA).

### Ribonuclease protection assay (RPA)

Total RNA was isolated from cell cultures of the clones (passages 5–7) using RNAeasy midi kits (Qiagen sciences, Maryland). RPA was performed using a mouse multi-probe BMP set (RiboQant system; BD Biosciences, Pharmingen, CA) that detected BMP -1, -2, -3, -3B, -4, -5, -6, -7, -8A and -8B, including L32 and GAPDH as internal controls. For each analysis, 15 µg of RNA was used. The protected fragments were separated on a 6% acrylamide, acrylamide/bis-acrylamide 19∶1, 7 M Urea and 1×TBE gel (Severn Biotech, UK). The gel was dried and exposed to a Biomax MR film (Kodak) for 1 to 4 days.

### Tumorigenicity in nude mice

Five clones from each cell line were subcutaneously inoculated in female Balb/c nu/nu mice (6 weeks; Bomholtgaard, Denmark) [16]. The cells (passages 4–9) were suspended in 100 µl PBS and were inoculated subcutaneously in the flank of the animal. If no tumor formation was observed after one month in any of the mice injected with the same clone, the group received a second injection in the other flank. Mice injected with the same clone but lacking sign of tumor growth one month after the second injection received a final injection (1×10^7^ cells in 100 µl PBS) subcutaneously in the lumbar region. The animal experiments were performed in accordance with protocols approved by the local ethical committee, approval number C135/3.

### Immunohistochemistry

Tissues were fixed in 4% buffered formalin. Bone tissues were decalcified and embedded in paraffin. A tissue microarray (TMA) [18] was prepared with the tumors generated from the CMT-U353 B clones. Two ø 1 mm punches were manually obtained from each tumor, transferred into one recipient TMA (Beecher Instruments, Silver Spring, MD, California), and baked in 60°C in 1 h. The TMA was sectioned (Microm HM 355 S; Microm, Walldorf, Germany) and put on Superfrost®Plus Gold slides (Menzel GmbH & Co KG, Braunschweig, Germany). Other tumor and control tissues without osseous areas, were put on Superfrost®Plus slides (Menzel GmbH & Co KG, Braunschweig, Germany). The 5 µm sections were baked in 37°C overnight and 1 h in 60°C. For antigen retrieval, the slides were placed in 0.02 M citrate buffer, pH 6.0 (PS1 antibody) or Target Retrieval Solution (Dako, Glostrup, Denmark) and boiled in a decloaking chamber (Biocare Medical). The following antibodies were used: a polyclonal antibody against phosphorylated Smad-1/5 (PS1)[19], a BMP-6 antibody (ab15640, Abcam, Cambridge, UK) and a polyclonal BMP-2/4 antibody (AF355, R&D systems, Europe Ltd, Abingdon, UK). The slides were incubated in 1% H_2_O_2_ in methanol for 30 min at room temperature prior to the BMP-6 antibody incubation. All slides were incubated overnight with the primary antibody at +4°C. For the stainings, the ABC-Elite system (Vector laboratories, CA, USA) and NovaRED (Vector laboratories) were used. To test the specificity of the PS1 antibody, a blocking peptide was used [19]; an excess of peptide (1.2 ng/µl) was incubated for 7 h with PS1 at 4°C before being incubated with the tissue sections. For every immunohistochemical analysis, positive and negative controls were used. Canine mammary gland, as well as the human breast cancer cell line MDA-MB-231 were used as positive controls for the BMP-6 antibody and canine rib joint was used as positive control for the BMP-2/4 antibody. For polyclonal antibodies, Tris buffered saline was used as negative control. As negative control for the monoclonal BMP-6 antibody, unspecific antibodies of IgG_1_ isotype were used.

### In vitro BMP stimulation

Canine mammary tumor cell clones and as a positive control HTh 74 cells, an anaplastic thyroid carcinoma cell line [20] were stimulated with BMP-2. Cells (80–100% confluent) were incubated overnight in RPMI 1640 (canine clones) or Eagle's minimal essential medium (HTh 74) with 0.5% fetal bovine serum. Subsequently, cell cultures were stimulated with 250 ng/ml BMP-2 (Peprotech, London, UK) in PBS/1.3% BSA for 1 h (37°C). Negative controls were incubated with PBS/1.3% BSA.

### Western blot

Cells were solubilized in cell lysis buffer (1% Triton X-100, 150 mM NaCl, 10 mM Tris-HCl pH 7.4, 1 mM EGTA, 1 mM EDTA and 0.5% NP-40) containing protease and phosphatase inhibitors (35 ng/ml phenylmethylsulfonyl fluoride (PMSF), 1 mM Na_3_VO_4_ and 1.4 µg/ml aprotinin). Lysates were incubated 30 min on ice and centrifuged 15 min at 15,000 x g (4°C). The samples were separated by gradient SDS-PAGE followed by transfer nitrocellulose filters. Filters were blocked with 5% BSA in TBS-T (10 mM Tris-HCl, pH 7.7, 0.15 M NaCl, 2% Tween-20) overnight. The filters were probed with antibodies to: Smad-5 [21], phosphorylated Smad-1/5 (PS1) (kind gift from Aristidis Moustakas, LICR, Uppsala, Sweden), Chordin-like 1 (MAB1808, R&D systems Europe Ltd, Abingdon, UK), p38α (#9217, Cell Signaling Technology, Beverly, MA), phosphorylated p38 (Phospho-p38 #9216, Cell Signaling Technology, Beverly, MA) and Smad-7 (kind gift from Maréne Landström, LICR, Uppsala, Sweden). The human prostate cancer cells PC-3U (kind gift from Maréne Landström, LICR, Uppsala, Sweden) were used as positive control for the P-p38, p38 and Smad-7 experiments. For the PS1 antibody, the specificity was verified by peptide blocking [19] (1.2 ng/µl of peptide; overnight incubation) before incubation with filters. Polyclonal α-Smad-5 antibody [21] and β-Actin (A5441, Sigma-Aldricht, St Louis, MO) were used as loading controls. Filters were incubated with antibodies at room temperature (1 h). HPR-conjugated anti-rabbit Ig (GE Healthcare, UK) was used as secondary antibody and a commercial kit, Super Signal®, with enhanced chemiluminescence (ECL) (Pierce, Rockford, IL) was used to visualize the results. Filters were stripped with a buffer containing 100 mM β-mercaptoethanol, 2% SDS and 62.5 mM Tris-HCl, pH 6.7.

## Results

To study the BMP expression profile in mammary tumors of different types, cell lines were established from a spindle cell tumor (CMT-U309), an osteosarcoma (CMT-U353B) and a scirrhous carcinoma (CMT-U353 H4) of canine origin. Subsequently, as detailed in Table 1, a number of clones were established from the parent cell lines.

**Table 1:** Tumors from cloned cell lines CMT-U309, CMT-U353 B and CMT-U353 H4 inoculated in mice.

Clone identity		Tumor take	Tumor type
CMT-U309 clone	1	0/5	No tumors formed
	2	0/5	No tumors formed
	4	3/5	Spindle-cell tumors (2), Spindle-cell tumor with bone formation (1)
	A5	0/5	No tumors formed
	C6	3/5	Spindle-cell tumors with bone formation (2), Spindle-cell tumor (1)
CMT-U353 B clone	1	5/5	Osteosarcomas
	2	4/5	Osteosarcomas
	3	0/5	No tumors formed
	6	3/5	Spindle-cell tumors
	7	4/5	Osteosarcomas
CMT-U353 H4 clone	5	3/5	Spindle-cell tumors (2), Fibroma durum (1)
	6	4/5	Spindle-cell tumors
	9	1/5	Anaplastic tumor
	10	4/5	Spindle-cell tumors
	12	1/5	Spindle-cell tumor

**Figure 1:**
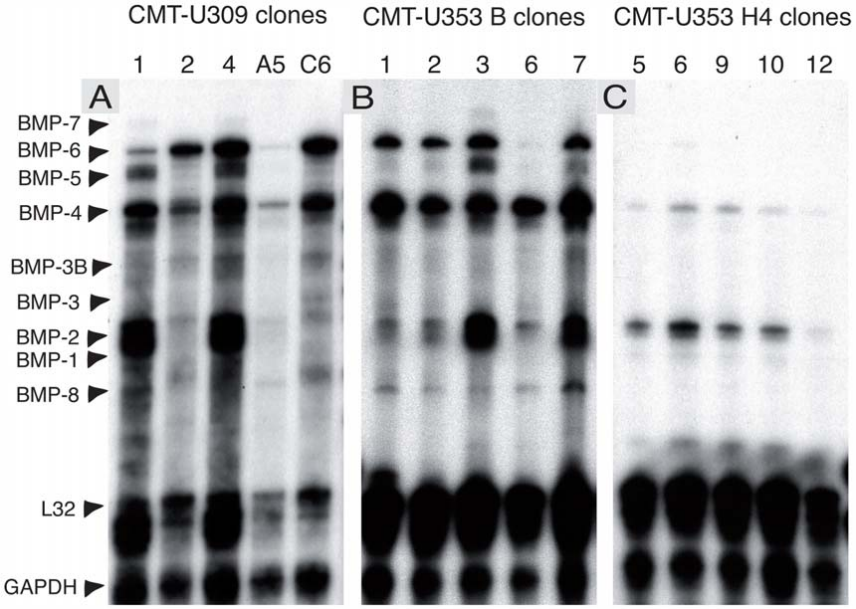
RPA analysis of expressed BMP mRNA in canine mammary clones. (A) Spindle cell clones (CMT-U309), (B) osteosarcoma clones (CMT-U353 B) and (C) scirrhous carcinoma clones (CMT-U353 H4).

RPA analysis showed that the osteosarcoma clones all expressed high levels of different BMPs (Figure 1B). In particular, high levels of BMP-4 were seen. Moreover, all of the clones, except CMT-U353B clone 6, expressed high levels of BMP-6 while BMP-2 expression varied among the osteosarcoma clones. Also BMP-5 was unevenly expressed between the clones (Figure 1B). BMP-8 expression levels were low but similar in all osteosarcoma clones. BMP-1 and -3 expression was not detected in any of the clones, whereas low levels of BMP-7 appeared to be expressed by clone 3. Because the primary spindle cell tumor did not form bone [17], it may be expected that the clones derived from it did not express high levels of BMPs. However, as shown in Figure 1A, we detected expression of BMP-2, -4, -5, and -6, although the expression profile and levels of expression varied among the different spindle cell clones. Notably, one of the spindle cell clones (clone A5) showed very low levels of BMP expression, although BMP-4 was detected. Since scirrhous carcinoma does not form bone, we expected low levels of BMP expression. Indeed, the various carcinoma clones expressed BMPs at low levels (Figure 1C).

We next asked how the various BMP expression patterns correlate with formation of tumors *in vivo*. Clones were inoculated into nude mice and tumors were allowed to form (Table 1). Notably, several of the clones did not produce tumors *in vivo*: CMT-U309 (clones 1, 2, A5), CMT-U353B (clone 3). Inoculation of spindle cell clones (CMT-U309) gave rise to spindle cell tumors and, noteworthy, bone formation was seen in half of the cases (Table 1). Interestingly, the CMT-U309 clones, 4 and C6, that formed bone tumors in the mice expressed BMP-6 (Figure 1). Inoculation of osteosarcoma clones (CMT-U353 B) produced osteosarcomas but, in one case (clone 6), spindle cells tumors were instead generated. Finally, inoculation of carcinoma clones (CMT-U353 H4) resulted in formation of either spindle cell-, fibroma durum- or anaplastic tumors, and in no case was bone formation seen (Table 1).

**Figure 2:**
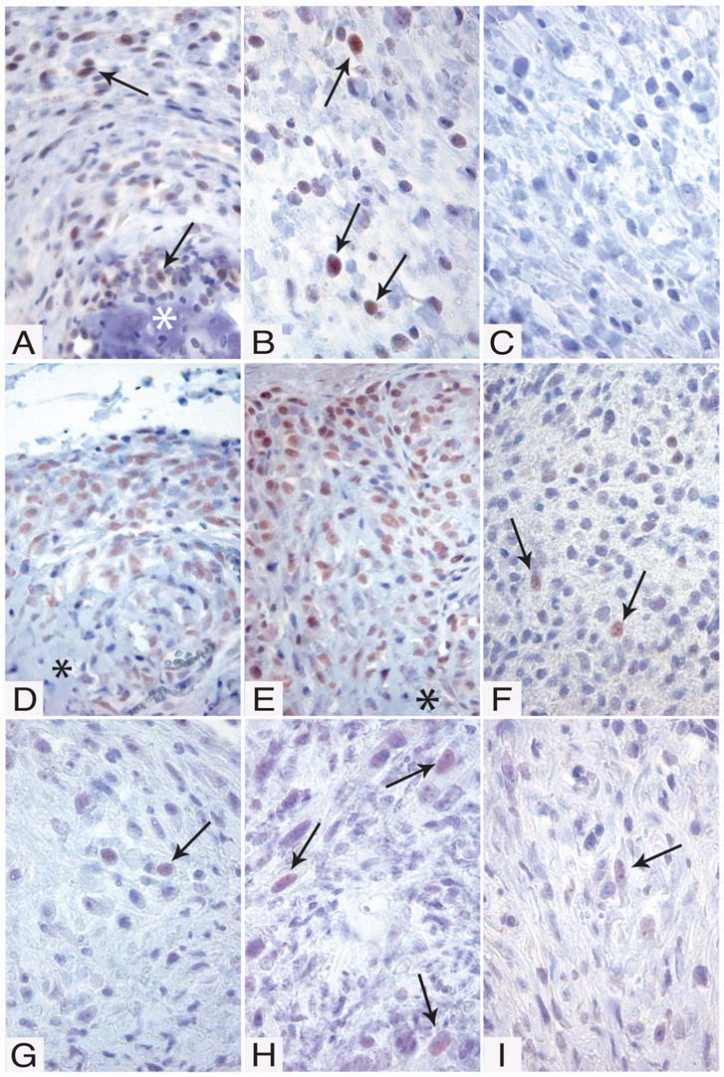
Immunohistochemical staining for phosphorylated Smad-1 and –5 (P-Smad-1/5). (A–C) Tumors generated by clones from the cell lines CMT-U309 (spindle cell); (D–F) CMT-U353 B (osteosarcoma); (G–I) CMT-U353 H4 (scirrhous carcinoma). (A–C) Spindle cell tumors generated by CMT-U309, clone C6. (A) Spindle cell tumor with an area of formed bone (*). Cells adjacent to the bone area and spindle cells with a distance to the formed bone showed clear positive staining (arrows). (B) Spindle cell tumors with strongly positive cells evenly distributed in the tumor (arrows). (C) Antibody specificity test: the signal was abolished when the antibody was incubated with blocking peptide. (D–E) Osteosarcomas formed by CMT-U353 B, clone 2 (D) and 7 (E). P-Smad-1/5 was detected in the cell dense border of the osteosarcomas. Cells closer to the centre of the tumor produced osteoid (*). (F) Spindle cell tumor generated by CMT-U353 B, clone 6. Bone formation was not seen in any tumors from this clone. P-Smad-1/5 was detected at lower levels than in tumors from clone 2 and 7, and was evenly distributed in the tumors (arrows). (G–H) Spindle cell tumors generated by clones from the scirrhous carcinoma: CMT-U353 H4, clone 6 (G), clone 9 (H) and clone 10 (I). Evenly distributed cells positive for P-Smad-1/5 were seen in the tumors (arrows). Note that positive cells show a predominantly nuclear staining.

In the next set of experiments we investigated whether the BMP expression was reflected by activated BMP signaling pathways, by analyzing for Smad-1/5 phosphorylation [6]. For this purpose, an antibody that recognizes both phosphorylated Smad-1 (P-Smad-1) and P-Smad-5 was used. As shown in Figure 2A, tumors derived from spindle cell clones were positive for P-Smad-1/5, particularly in the vicinity of bone (Figure 2A and results not shown), but also in the spindle cell area (Figure 2B). Notably, P-Smad-1/5 was primarily located in the nucleus, in agreement with translocation of P-Smad-1/5 in to the nucleus [6]. The experimental tumors from the osteosarcoma clones showed strong staining for P-Smad-1/5, in particular at the edges of the tumor. The degree of P-Smad-1/5 staining varied among the different clones, with clones 2 (Figure 2D) and 7 (Figure 2E) showing stronger staining than clone 6 (Figure 2F). All of the tumors derived from the scirrhous carcinoma clones were only weakly positive for P-Smad-1/5 (Figure 2G-I).

**Figure 3:**
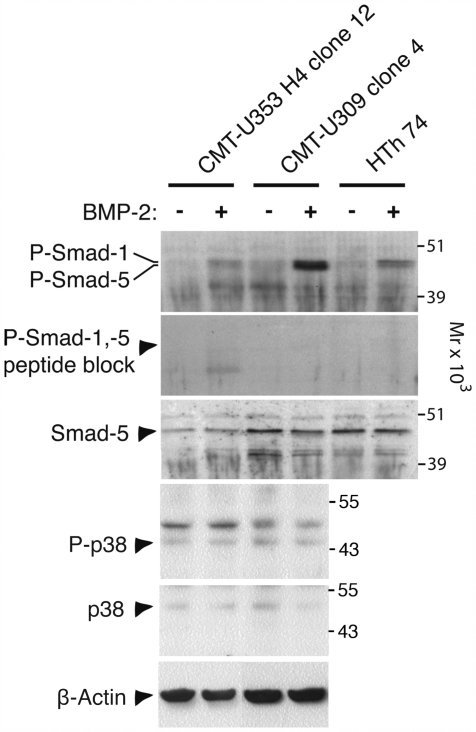
The effect of BMP-2 on phosphorylation of Smad-1, Smad-5 and p38 in mammary tumor clones. CMT-U353 H4 (clone 12), CMT-U309 (clone 4) and HTh 74 (positive control for Smad-1/5 phosphorylation) cells were stimulated with BMP-2 (as indicated) in vitro and phosphorylation of Smad-1/5 (P-Smad-1, -5) and p38 (P-p38) was analyzed by Western blot. Immunoblot analysis for total Smad-5, total p38 and β-Actin was performed as loading controls.

To further verify that the Smad-1/5 pathway is activated in the mammary tumors, tumor cells were stimulated with BMP followed by detection of P-Smad-1/5 by Western blot analysis. One clone showing high levels of BMP expression (CMT-U309, clone 4; see Figure 1) and one clone showing low BMP expression (CMT-U353 H4, clone 12; see Figure 1) were stimulated with BMP-2. As a positive control, the human anaplastic thyroid carcinoma cell line HTh 74, known to respond to BMP stimulation [22], was included. As shown in Figure 3, all of the three cell lines responded to BMP-2 stimulation by Smad 1/5 phosphorylation. Strikingly, the clone with the highest level of BMP expression (CMT-U309, clone 4) showed a much more robust response as compared to the low BMP-expressing clone (CMT-U353 H4, clone 12). There is also the possibility that the stimulation with BMP-2 may activate the p38 pathway, which would be reflected by phosphorylation of p38 [23], [24]. To address this possibility, BMP-2-stimulated CMT-U309, clone 4 and CMT-U353 H4, clone 12 were also analyzed for the levels of phosphorylated p38 (P-p38). As shown in Figure 3 these clones indeed expressed p38 protein and it was also evident that basal phosphorylation of p38 was present. However, BMP-2 stimulation did not cause an increase in the level of P-p38. Together, these data suggest that BMP-2 preferentially activates the Smad-1/5 pathway in CMT-U309, clone 4 and CMT-U353 H4, clone 12, causing minimal activation of p38.

**Figure 4:**
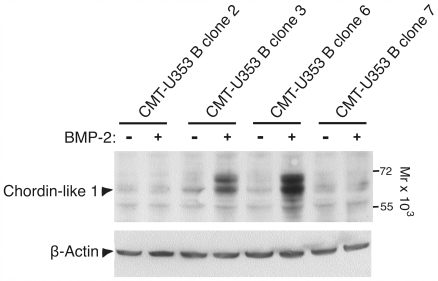
Expression of Chordin-like 1 protein in response to BMP-2 stimulation analyzed by Western blot. CMT-U353 B (clones 2, 3, 6 and 7) were either non-stimulated or stimulated with BMP-2 as indicated. β-Actin was used as loading control.

The activity of BMPs is stringently regulated by BMP antagonists such as Chordin-like 1 [25]. A possible explanation for the different ability of the various clones to generate bone-containing tumors *in vivo* could thus be related to differences in Chordin-like 1 expression. To address this possibility, we therefore assessed the levels of Chordin-like 1 protein in various clones, and if the levels were affected by BMP-2 stimulation. As shown in Figure 4, the levels of Chordin-like 1 in response to BMP-2 stimulation varied markedly among the clones. Strikingly, the Chordin-like 1 levels were considerably higher in non-tumor forming clones (CMT-U353 clone 3) and in a clone that formed tumors without bone (CMT-U353 B clone 6) than in bone-forming clones (CMT-U353 B clones 2 and 7), (see Table 1). Hence, these data are compatible with a scenario in which the bone-generating capacity of the respective clones could be related to their expression of BMP antagonists.

Further, we have analyzed Smad-7 protein expression, an inhibitory Smad. The results showed clear expression of Smad-7 in all clones tested. However, the expression levels were very similar among the different clones, and there was no correlation between basal levels of Smad-7 expression and sensitivity to BMP-stimulation or bone formation (not shown).

**Figure 5:**
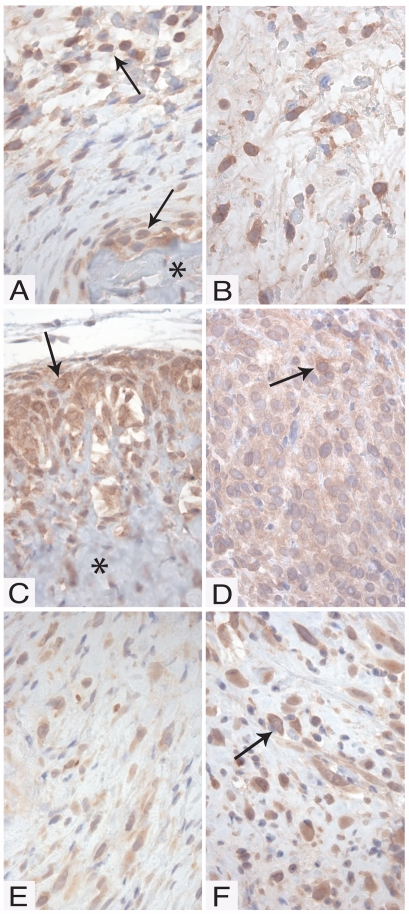
Immunohistochemical analysis for BMP-6. BMP-6 staining was performed in tumors generated by clones from the CMT-U309 (spindle cell), CMT-U353 B (osteosarcoma) and CMT-U353 H4 (scirrhous carcinoma) cell lines. (A–B) Spindle cell tumors generated by CMT-U309, clone C6. (A) Spindle cell tumor with an area of formed bone (*). Cells adjacent to the bone area and spindle cells further away from the formed bone showed strong positive cytoplasmic staining (arrows). (B) Spindle cell tumors with cells strongly positive for BMP-6, evenly distributed in the tumors. (C) Osteosarcoma formed by CMT-U353 B, clone 2. BMP-6 was detected in the cell dense border of the osteosarcoma and some cells showed membranous accentuation of the staining (arrow). (D) Spindle cell tumor generated by CMT-U353 B, clone 6. Faint staining for BMP-6 was seen throughout the tumors, with only a few cells with stronger staining and clear cytoplasmic positivity (arrow). (E–F) Spindle cell tumors generated by CMT-U353 H4, clone 6 (E) and 9 (F) were positive for BMP-6. Tumor cells showed cytoplasmic positivity and some cells had membranous accentuation of the staining (arrow).

Previous studies indicate that, out of the different BMPs, BMP-6 may hold a key position in a number of processes, including bone formation [26] and wound healing [27]. Next, we therefore analyzed the various tumors for presence of BMP-6 protein. Tumors derived from spindle cell clones were strongly positive for BMP-6 (Figure 5A–B), in agreement with the high mRNA levels for BMP-6 in the corresponding clones (see Figure 1). Notably, the staining was particularly strong in the vicinity of bone tissue and also in the spindle cells forming the major part of the tumor. Also tumors formed from a high BMP-6-expressing osteosarcoma clone (CMT-U353 B, clone 2; see Figure 1) showed strong staining for BMP-6, with particularly strong staining at the edge of the tumor (Figure 5C). Interestingly, the staining was accentuated at the cell membranes (Figure 5C; arrow). In contrast, when tumors from an osteosarcoma clone with low expression of BMP-6 mRNA (CMT 353 B, clone 6; see Figure 1) were analysed, only weak, diffuse BMP-6 staining was observed (Figure 5D). Unexpectedly, tumors from scirrhous carcinoma clones, i.e. clones showing low levels of BMP-6 mRNA expression *in vitro* (see Figure 1) and a low degree of Smad-1/5 pathway activation (see Figure 2G-I), were strongly positive for BMP-6 protein (Figure 5E-F).

**Figure 6:**
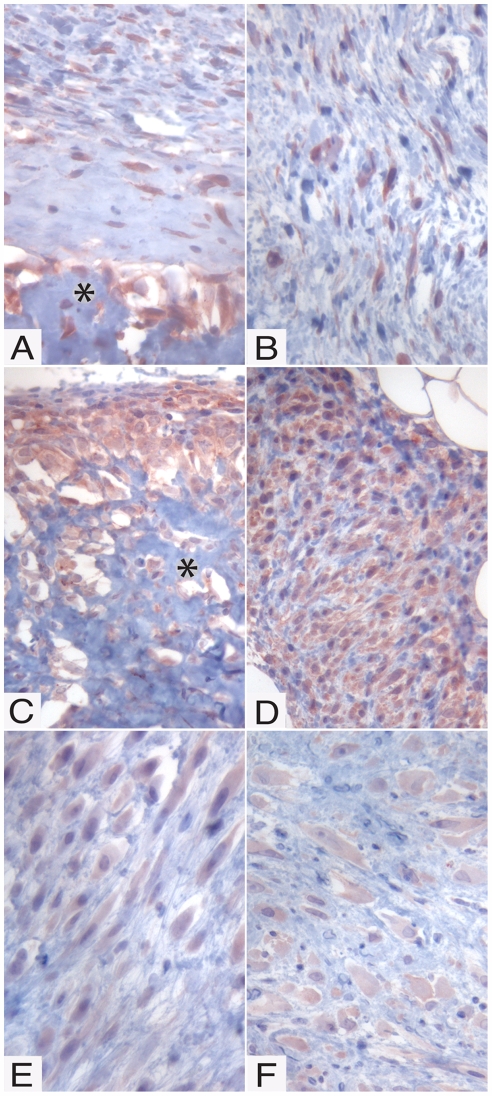
Immunohistochemical analysis for BMP-2/4. Staining for BMP-2/4 was performed in tumors generated by clones from the CMT-U309 (spindle cell), CMT-U353 B (osteosarcoma) and CMT-U353 H4 (scirrhous carcinoma) cell lines. (A–B) Spindle cell tumors generated by CMT-U309, clone C6. (A) Spindle cell tumor with an area of formed bone (*). Cells adjacent to the bone area and spindle cells further away from the formed bone showed strong positive cytoplasmic staining. (B) Spindle cell tumors with cells strongly positive for BMP-2/4, evenly distributed in the tumors. (C) Osteosarcoma formed by CMT-U353 B, clone 2. BMP-2/4 was detected in the cell dense border of the osteosarcoma as well as in cells adjacent to bone (*). (D) Spindle cell tumor generated by CMT-U353 B, clone 6. Strong staining for BMP-2/4 was seen throughout the tumors. (E–F) Spindle cell tumors generated by CMT-U353 H4, clone 6 (E) and 9 (F) stained less intensely for BMP-2/4.

Since the RPA analysis revealed strong expression of BMP-2 and -4 in a number of clones, it was of interest to assess whether the in vitro expression patterns of these BMPs were reflected by their presence in the tumors formed *in vivo* from the respective clones. As shown in Figure 6A and B, the tumor formed by CMT U309, clone 6, i.e. a clone highly expressing BMP-4 in vitro (see Figure 1), was strongly positive for BMP-2/4. Strong positivity was also seen in tumors formed from CMT-U353 B, clones 2 (Figure 6C) and 6 (Figure 6D), i.e. clones with high expression of BMP-4 (see Figure 1). Similar to the staining for BMP-6 (see Figure 5C), strong staining for BMP-2/4 was seen particularly in tissue adjacent to bone (Figure 6C). It is also clear that tumors formed from CMT-U353 H4, clones 6 and 9 stained less intensely for BMP-2/4, in agreement with a low expression of BMP-4 and only modest expression of BMP-2 as judged by the RPA analysis (see Figure 1).

## Discussion

This study is, to our knowledge, the first in which the expression profile for BMPs in different types of canine mammary tumors is determined in concert, although some studies have addressed the expression of individual BMPs in selected types of mammary tumors and cell lines [12], [28]–[31]. Moreover, previous studies have mostly addressed the BMP expression in primary tumors and it is therefore difficult to ascertain whether the specific BMP expression pattern is a result of contribution from a number of different cell types present in the primary tumor, or if the total BMP expression pattern is a result of one single type of cell. To specifically address the latter issue we here studied the BMP expression profile in cell clones derived from the respective type of tumor and, to ensure that the expression pattern found mimics that of the original cell from the primary tumor, BMP expression patterns were studied at low cell passage numbers.

We show that each of a number of clones from mammary tumors expresses a panel of different BMPs. We also show that the BMP expression profile is highly variable, both as regards which BMPs that are expressed and also as regards the levels of expression for the various BMPs. Hence, the total BMP expression pattern within a tumor is probably the result of distinct contributions from several types of cells types found within an individual tumor. When comparing the BMP expression profiles of the different types of mammary tumor clones, we were not able to see any clear cut differences in either BMP expression repertoire or expression levels when comparing clones from the spindle cell and osteosarcoma. In contrast, we show that the scirrhous carcinoma clones express considerably lower levels of BMP transcripts than the spindle cell and osteosarcoma clones. Moreover, the carcinoma cells displayed a more limited repertoire of BMPs being expressed with, for example, undetectable BMP-5, -6, -7 and –8 expression. Notably, the expression of the control gene GAPDH was highly consistent among all clones whereas the expression of the other used control gene, L32, was consistent among the osteosarcoma and carcinoma clones but was less consistent among the spindle cell clones.

An important issue is whether the BMP expression pattern/level of the various clones can be correlated with tumorigenicity. We show that 3 of the clones derived from the spindle cell tumor (CMT-U309, clones 1, 2, A5) were unable to generate tumors *in vivo*, after inoculation in nude mice. Out of these, clone 1 and 2 expressed rather high levels of BMPs and it is therefore not possible to correlate their lack of tumorigenicity with a low general expression of BMPs. Further, the only clone derived from the osteosarcoma not being able to form tumors *in vivo* (clone 3) expressed very high levels of BMPs (BMP-2, -4, -5, -6) *in vitro*, thus further supporting the notion that the BMP expression profile of the inoculated clones is not a major determinant for tumorigenicity. On the other hand, since the latter clone expressed high levels of the BMP antagonist, Chordin-like 1, its lack of tumorigenicity may be related to blocked BMP-mediated pathways.

Considering the strong implication of BMPs, in particular BMP-6, in both physiological and tumorous bone formation [8], [9], [29], [30], [32], [33], it is also of interest to assess whether the expression pattern/levels of BMPs can be linked to bone formation in tumors. Interestingly, we found that all of the clones that gave bone formation expressed high levels of BMP-6 mRNA, whereas all of the clones that failed to generate bone were low producers of BMP-6. BMP-2 was expressed at high levels in some bone-producing clones (CMT-U309, clone 4; CMT-U353 B, clone 7) but was barely detectable in others (e.g., CMT-U309, clone C6), suggesting that BMP-2 is not of major importance in tumorous bone formation. This notion is also supported by a study in which delivery of BMP-2 via an adenoviral vector failed to give bone formation in nude rats [32]. BMP-4 was highly expressed in all bone-forming clones, but was also expressed at rather high levels in a non-bone generating osteosarcoma clone (clone CMT-U353 B, clone 6) as well as in all of the carcinoma clones. Hence, we were not able to see a clear link between BMP-4 expression levels and bone formation. Together, our results are thus compatible with a scenario in which BMP-6 plays a major role in the formation of bone in mammary tumors, a finding that is in line with previous other reports [8], [32].

When assessing the levels of BMP-6 protein in the various tumors we found that tumors derived from high BMP-6-expressing spindle cell and osteosarcoma clones, as expected, were strongly positive for BMP-6 protein. Further, tumors formed from the low BMP-6-producing osteosarcoma clone (CMT-U353 B, clone 6) were only weakly positive for BMP-6 protein. In contrast, we unexpectedly found that also tumors derived from the carcinoma clones, i.e. low producers of BMP-6, were positive for BMP-6 protein. We cannot with certainty explain this apparent discrepancy, although we favor the possibility that the BMP-6 protein expression in the carcinoma clones may have been upregulated during the *in vivo* inoculation process. On the other hand, although BMP-6 protein is present, the BMP pathway does not appear to be functional, as shown by the low degree of Smad-1/5 phosphorylation.

In summary, our study shows that BMPs are highly expressed in cell clones derived from various mammary tumors, thus implicating BMPs in breast cancer. However, it remains to be determined if the BMP expression profile of the various clones exactly matches the BMP profile of the corresponding formed tumors.
